# Author Correction: Molecular insights and antibody response to Dr20/22 in dogs naturally infected with *Dirofilaria repens*

**DOI:** 10.1038/s41598-024-65401-w

**Published:** 2024-06-24

**Authors:** Mateusz Pękacz, Katarzyna Basałaj, Daniel Młocicki, Maciej Kamaszewski, Elena Carretón, Rodrigo Morchón, Marcin Wiśniewski, Anna Zawistowska-Deniziak

**Affiliations:** 1 Division of Parasitology, Department of Preclinical Sciences, Institute of Veterinary Medicine, Warsaw University of Life Sciences-SGGW, 02-786 Warsaw, Poland; 2 Museum and Institute of Zoology, Polish Academy of Sciences, 00-818 Warsaw, Poland; 3 Department of General Biology and Parasitology, Medical University of Warsaw, 02-004 Warsaw, Poland; 4 Department of Ichthyology and Biotechnology in Aquaculture, Institute of Animal Sciences, Warsaw University of Life Sciences-SGGW, 02-786 Warsaw, Poland; 5 Internal Medicine, Faculty of Veterinary Medicine, University of Las Palmas de Gran Canaria, Campus Arucas, Arucas, 35413 Las Palmas, Spain; 6 Zoonotic Diseases and One Health Group, Faculty of Pharmacy, University of Salamanca, Campus Miguel Unamuno, 37007 Salamanca, Spain; 7 Department of Immunology, Institute of Functional Biology and Ecology, Faculty of Biology, University of Warsaw, 02-095 Warsaw, Poland

Correction to: *Scientific Reports* 10.1038/s41598-024-63523-9, published online 05 June 2024

The Funding section in the original version of this Article was incomplete. The Funding section now reads:

"This research was funded by National Centre for Research and Development, grant number 0106/L-9/2017. The publication was (co)financed by the Science Development Fund of the Warsaw University of Life Sciences—SGGW.”

The original Article has been corrected.

