# Supplementary material for: Molecular insights and antibody response to Dr20/22 in dogs naturally infected with Dirofilaria repens
Source: Sci Rep. 2024 Jun 5;14:12979. doi: 10.1038/s41598-024-63523-9 (PMC11153217; doi:10.1038/s41598-024-63523-9)
Supplement: Supplementary file 2 — Supplementary Figures. [file 41598_2024_63523_MOESM2_ESM.pdf]

Supplementary Material

ATGAACAAACTTTTCATAGTTCTTGGCTTAGTGATTCTTTCTGTTGCATTACCTTCTGCATCAGAATCAGAAGAAGA
GGTAATTTCTTCAGTTCAGTTTCTCAACTTTTAATAATATCAAATTAAAAGTAATCAGCAACAGGAATAATGACCAA
ATAAGGAAGATAAGGAATATATATTGAAGAGAATACATGCTAATCAAGTGCACTACTTCTTGGATTGGAGAAAAA
GAAAAAGATTATGTTCAAGTCATTTTTTCGATCAAAATTTTCATAAAAGTATCTTTATGATTTCAGAGTGTATCTTTT
GAAGACAGCGACGAAAGTTATACAGAAGATGATGAAGGTCATAAAGAAGAACACAATGATCATGCAACTGAAG
ACGATGAATATGTAACTAAAGGAAAATTTGTTGAAAGTGATGGTAAGTAAGTCGTTTCAATCTTTCAAGTATTTAA
ATTCCTGAAAACTTCATTAAAACTTTAGTTCGCAACCATATGTGTTCTTCTATCCATCCTGCAGCGCTGTTCCGCTAC
TCGAAATATAATTTCGCTATAATATGTTTTAAATTTCTTCATTTAAATAATGCGATTCAATAACTTTCCCAATACATTAC
AGATGATTTCCAATATGGACTGATTCGCTTACATTTCATTTTAATTTCTTGCAGAATGAAACACTGCAAAACTCATG
AAGCTTGCTATGACCAACGTGAACCGCAATCGTGGTGCATATTGAAACCGCATCAATCATGGACGTAAGCACCTT
ATGATTATTCTAACGAAAAAAATAGTTTAGATAAGAATTTTCGATGCCTATTAATCAGAATTTCCGATTCCTATAAGT
CAGAATTTCCTTCTAAATCAATATCCATAAATGTCTATAAAATCCATAAATTTCGCAATAAAATTGAGAACTTTTTGT
CTTAGAAAAAGAGGTTGTTTCTGCGAATCAAAAAAGCATGCATGCGTTATCGAGCGGAAGAGCGGCGACAACTT
TGAATATTCATATTGCTCACCACGAAAAGACTGGCAGTGCTCATATGATTAA

Legend:

Exon1: 1-78 bp **(78 bp)**

Exon2: 296 - 422 bp **(127 bp)**

Exon3: 665 -755 bp **(91 bp)**

Exon4: 928 - 1,048 bp **(121 bp)**

STOP CODON

Supplementary Figure 1. Genomic DNA sequence of *dr20/22* with determined exons and introns.

```
                   1        10        20        30        40        50        60        70        80        90        100       110       120       130
                   |--------+---------+---------+---------+---------+---------+---------+---------+---------+---------+---------+---------+---------|
DI<MCP9263775.1>   MNKLFIFLGLVLLFVSSFSASESESKDVTFEESDEDDEED------EEKSEEQGNQSNEHHDHATEDDEYVTKGEFVESDGKMKHCESHEACYDQREPQSWCILKPHQSWTQRGCFCESKKHACVIERKS
 DI<AAC47031.1>    MNKLFIVLGLALLFVALPSASESQEETVSFEESDEDYEDDSEDQTKEEEHSKEEDRSEEHDDHSAEDDKFVTKGKFVESDGKMKHCKTHEACYDQREPQSWCILKPHQSWTQRGCFCESKKHACVIERKS
 DR<QHR84804.1>    MNKLFIVLGLVILSVAFPSASQSEEESVSFEDSDESYAED------------YEGHKEEHNDHATEDDEYVTKGKFVESDGKMKHCKTHEACYDQREPQSWCILKPHQSWTKRGCFCESKKHACVIERKS
      Consensus    MNKLFIvLGLvlLfVa.pSAS#S#ee.VsFE#SDEdye#D......ee.....e..s#EH.DHatEDDe%VTKGkFVESDGKMKHCktHEACYDQREPQSWCILKPHQSWTqRGCFCESKKHACVIERKS

                   131      140       150
                   |--------+---------|
DI<MCP9263775.1>   GDKLEYSYCSPRNNWQCSYD
 DI<AAC47031.1>    GDKLEYSYCSPRKNWQCSYD
 DR<QHR84804.1>    GDNLEYSYCSPRKDWQCSYD
      Consensus    GDkLEYSYCSPRk#WQCSYD
```

Supplementary Figure 2. Comparison of amino acid sequences of *D. repens* ALT (QHR84804.1) and two variants of *D. immitis* ALT (MCP9263775.1; AAC47031.1).

Supplementary Figure 3

2023-03-06 14hr 57min.scnDES20fresh

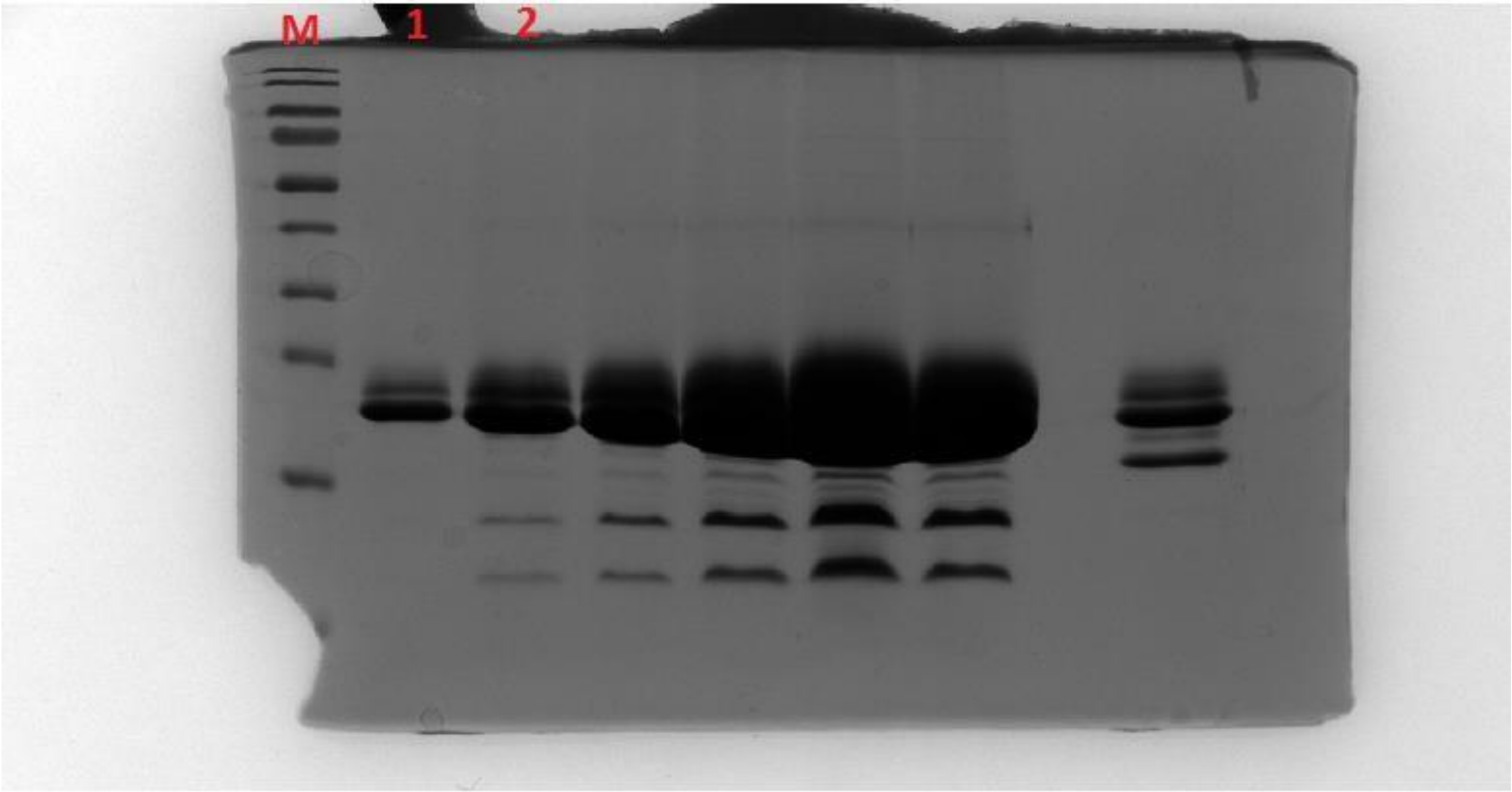

Figure 2A

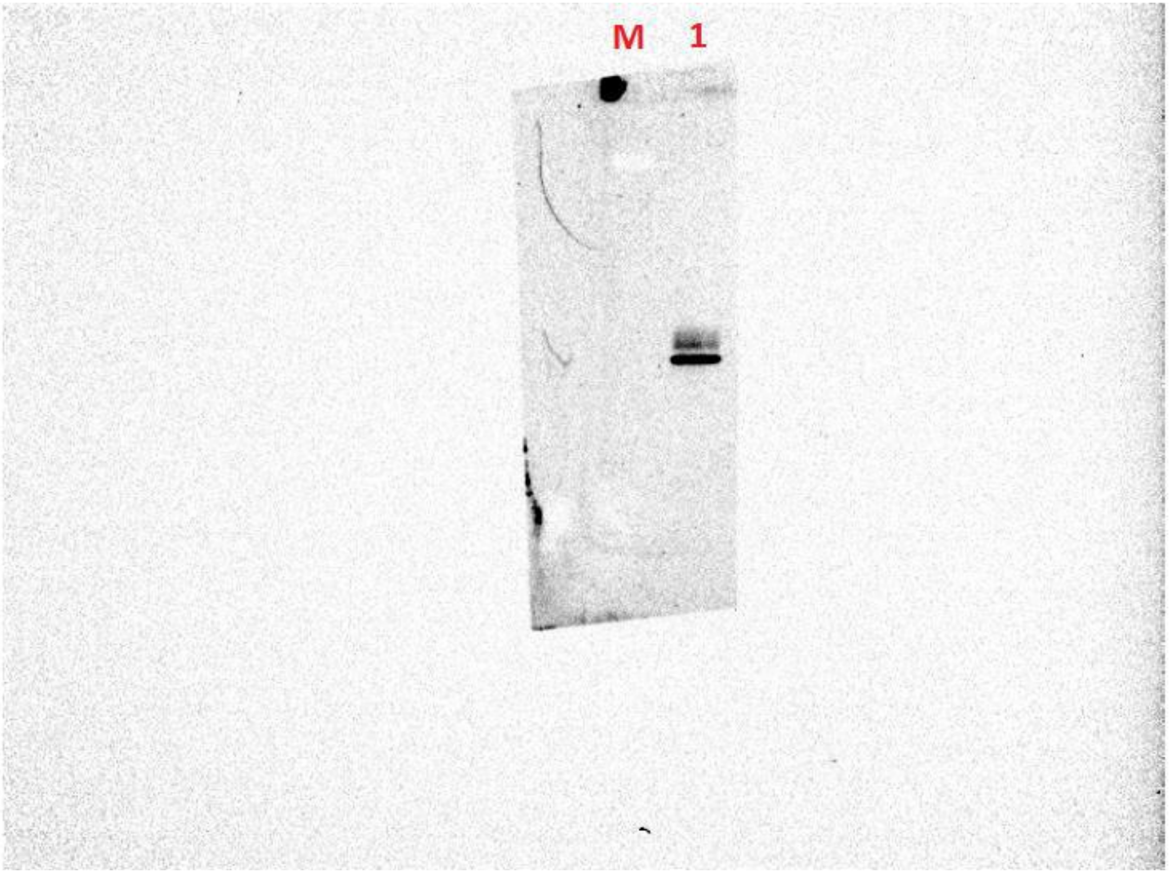

Figure 2B

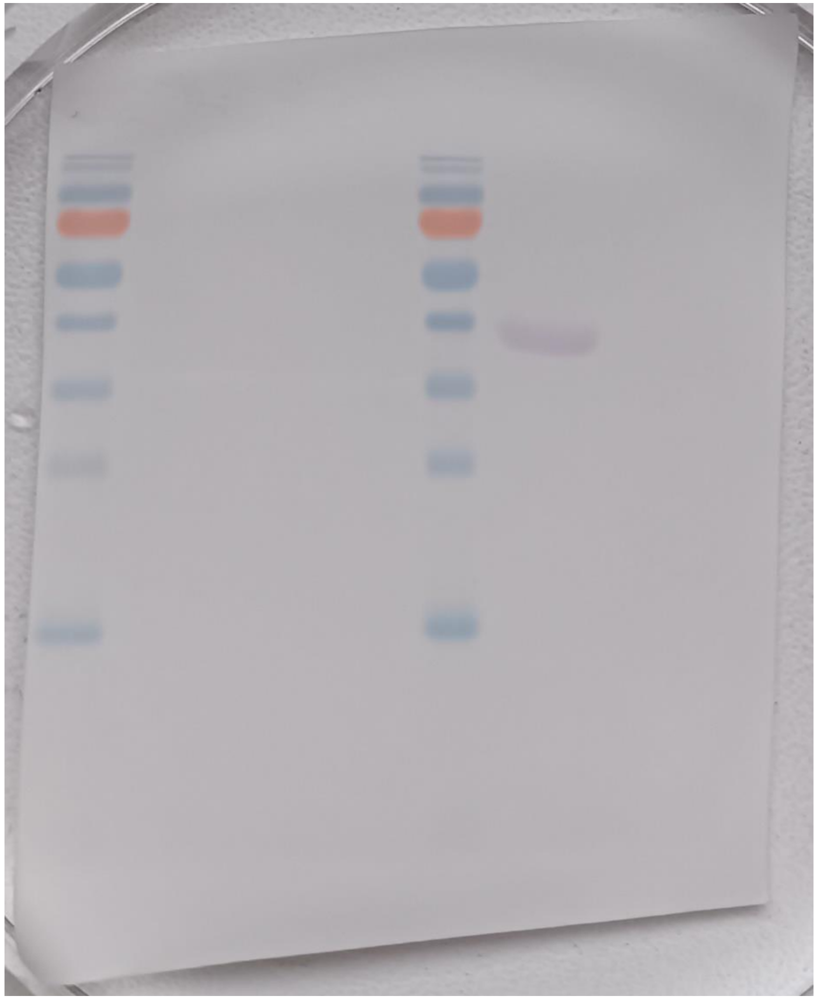

Figure 3

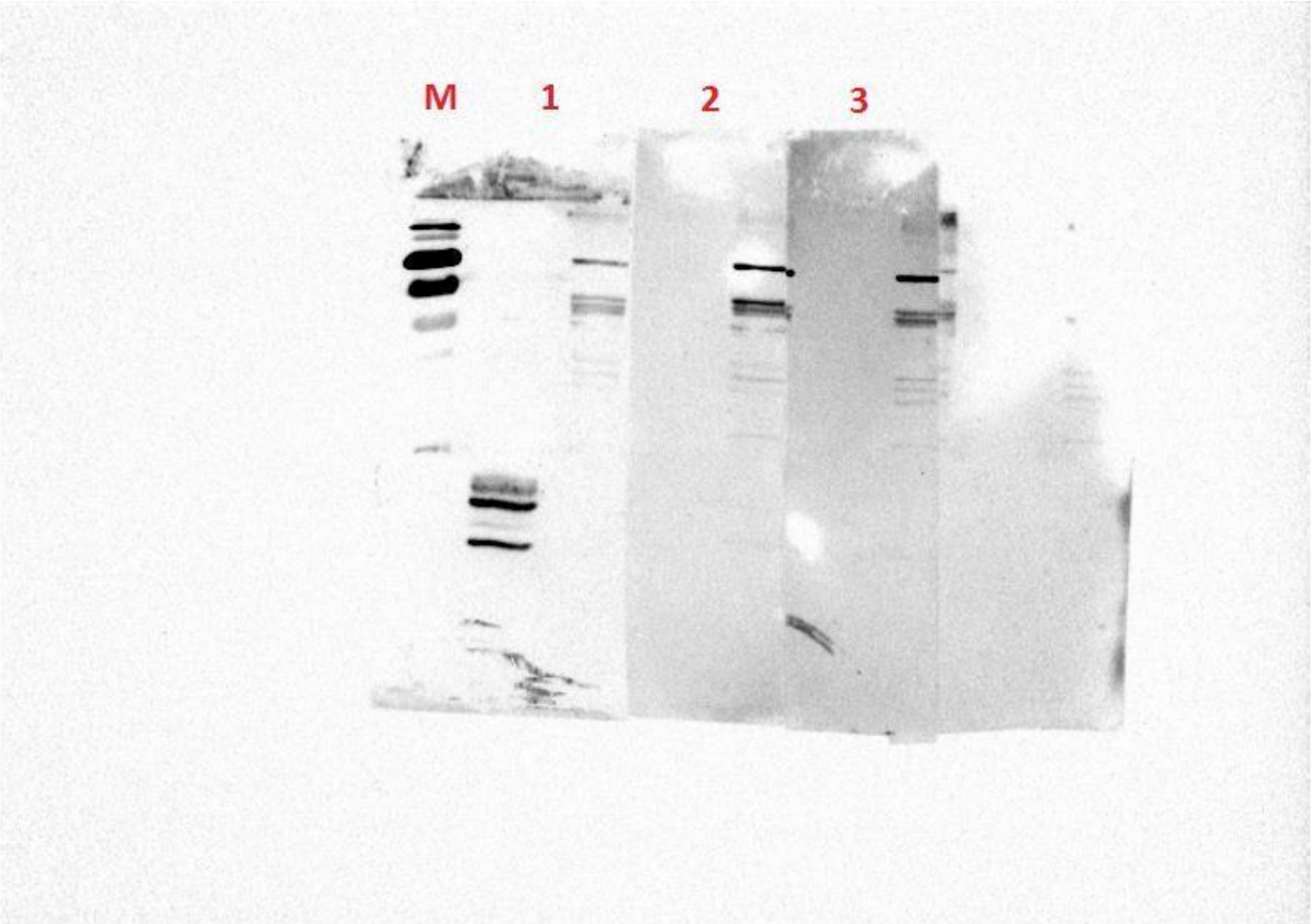

Figure 4

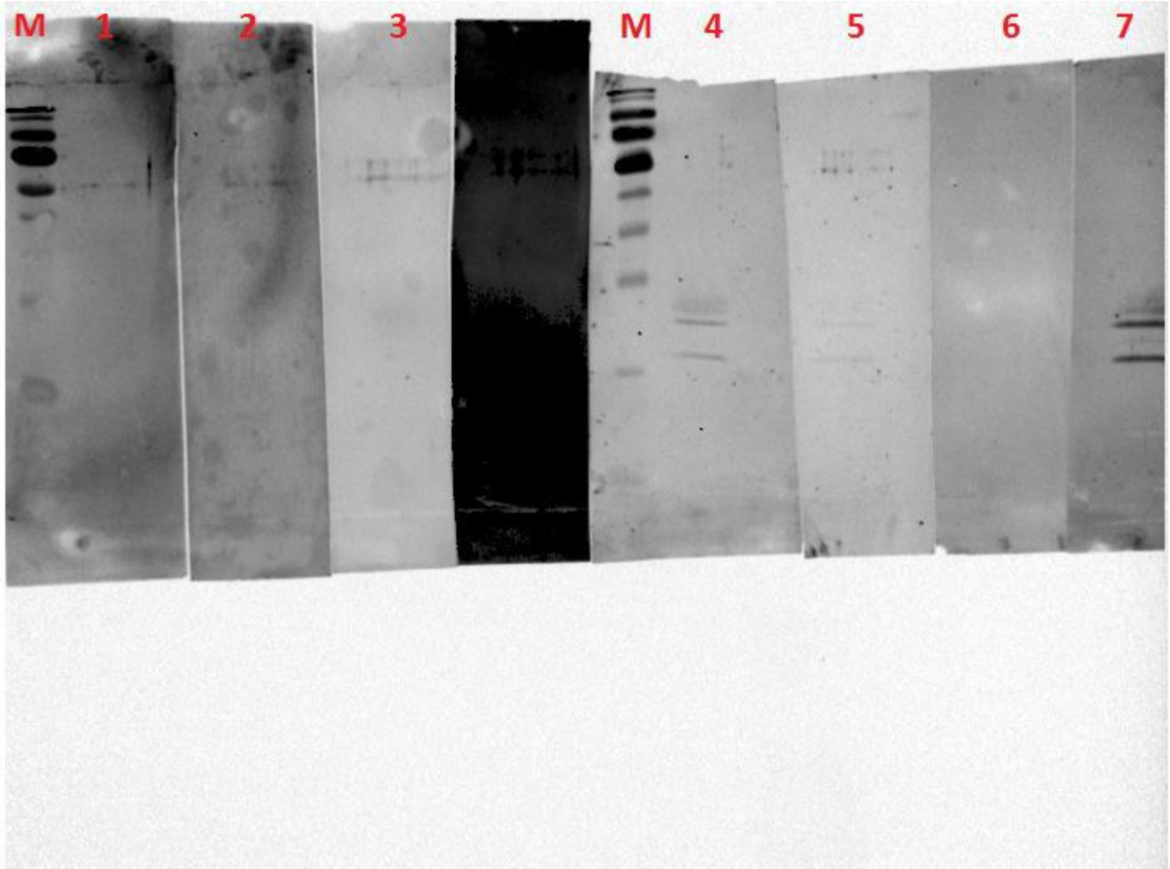

Figure 7
